# Noninvasive Prenatal Testing in Immunohematology—Clinical, Technical and Ethical Considerations

**DOI:** 10.3390/jcm11102877

**Published:** 2022-05-19

**Authors:** Jens Kjeldsen-Kragh, Åsa Hellberg

**Affiliations:** 1 Clinical Immunology and Transfusion Medicine, Office for Medical Services, Region Skåne, SE-221 85 Lund, Sweden; asa.hellberg@skane.se; 2 Department of Laboratory Medicine, University Hospital of North Norway, N-9019 Tromsø, Norway

**Keywords:** hemolytic disease of the fetus and newborn, fetal and neonatal alloimmune thrombocytopenia, noninvasive prenatal testing, polymerase chain reaction, next-generation sequencing, digital PCR, pregnancy, alloimmunization, cell-free fetal DNA, ethics

## Abstract

Hemolytic disease of the fetus and newborn (HDFN), as well as fetal and neonatal alloimmune thrombocytopenia (FNAIT), represent two important disease entities that are caused by maternal IgG antibodies directed against nonmaternally inherited antigens on the fetal blood cells. These antibodies are most frequently directed against the RhD antigen on red blood cells (RBCs) or the human platelet antigen 1a (HPA-1a) on platelets. For optimal management of pregnancies where HDFN or FNAIT is suspected, it is essential to determine the RhD or the HPA-1a type of the fetus. Noninvasive fetal RhD typing is also relevant for identifying which RhD-negative pregnant women should receive antenatal RhD prophylaxis. In this review, we will give an overview of the clinical indications and technical challenges related to the noninvasive analysis of fetal RBCs or platelet types. In addition, we will discuss the ethical implications associated with the routine administration of antenatal RhD to all pregnant RhD-negative women and likewise the ethical challenges related to making clinical decisions concerning the mother that have been based on samples collected from the (presumptive) father, which is a common practice when determining the risk of FNAIT.

## 1. Introduction

A core feature of the immune system is its ability to distinguish between self from nonself. This property allows the body to mount an immune response against foreign invaders such as bacteria, fungi, viruses, etc. while being tolerant of the body’s own cells and tissue. A disturbance of the immune system’s balance between recognizing self and nonself can lead to a state of immunodeficiency if the nonself is not properly recognized or autoimmunity if an immune response is mounted against the body’s own cells or tissue.

During normal pregnancy, the woman carries a fetus who has inherited half of its genome from the father. From an immunological point of view, is seems paradoxical that the woman’s immune system does not reject the fetus since it expresses numerous antigens inherited from the father, which are foreign to the woman. The pregnant woman’s immune system tolerates the fetus because numerous, primarily local, immunological mechanisms in the placenta prevent the maternal immune system from rejecting the fetus [1].

Fetal–maternal bleeding is a rather common event, particularly during the third trimester. If fetal blood cells entering the maternal circulation carry paternally inherited antigens that are foreign to the woman’s immune system, her body may mount an antibody response against these paternally inherited antigens. Antibodies of the IgG class can traverse the placenta and bind to the paternally inherited antigens on the fetal cells. These sensitized fetal blood cells are then removed by the mononuclear phagocyte system in the fetus.

Three distinct clinical conditions are associated with maternal antibodies against paternally inherited antigens on red blood cells (RBCs), platelets and neutrophile granulocytes and are known as hemolytic disease of the fetus and newborn (HDFN), fetal and neonatal alloimmune thrombocytopenia (FNAIT) and neonatal alloimmune neutropenia (NAIN), respectively. If untreated, HDFN may result in severe fetal anemia that may lead to hydrops fetalis and stillbirth. Postnatally, there is severe anemia, and jaundice may lead to severe neurological damage due to deposits of bilirubin in the grey matter of the central nervous system [2]. HDFN is most commonly caused by antibodies against the RhD antigen on RBCs. FNAIT may cause severe fetal thrombocytopenia, which may lead to intrauterine death or severe neurological damage due to intracranial hemorrhage (ICH) [3]. In Whites, the most common (around 80%) and the most severe cases of FNAIT are caused by antibodies against human platelet antigen 1a (HPA-1a) [3]. NAIN can be associated with severe neutropenia, which does not have any adverse consequences in the fetus, but may leave the newborn prone to infections in the neonatal period [4]. However, NAIN usually has a benign course as infections in the neonatal period can effectively be treated with antibiotics. For this reason, we will only discuss in this review the role of noninvasive prenatal testing in relation to anti-D-associated HDFN and anti-HPA-1a-associated FNAIT.

## 2. Hemolytic Disease of the Fetus and Newborn

### Clinical Considerations

Around five decades ago, HFDN was associated with significant mortality and morbidity. Hence, HDFN affected 150 per 100,000 births and was responsible for 10% of all perinatal deaths [2]. Within a few years after the implementation of RhD prophylaxis, there was a dramatic reduction both in the number of RhD-immunized women and in the number of HDFN cases in North America and Western Europe. Thus, RhD prophylaxis became one of the most effective immunological interventions in clinical medicine.

The RhD prophylaxis was based on the administration of a one single dose of hyperimmune anti-D IgG to all RhD-negative women within 72 h after delivery of an RhD-positive child and in relation to any event that is known to be associated with the risk of fetal–maternal bleeding. Despite this initial success, it became clear that a small proportion of RhD-negative women carrying an RhD-positive fetus still became RhD-immunized despite the administration of hyperimmune anti-D IgG after delivery. Most of these cases were due to fetal–maternal bleeding in the third trimester. This led to a modification of the RhD prophylaxis, which involved the administration of one or two doses of hyperimmune anti-D IgG in the third trimester in addition to a dose after delivery of an RhD-positive child. Although this routine was adopted by several countries such as the USA, Canada and the UK, there was one drawback: Since the fetus’s RhD type is not known, it is necessary to administer hyperimmune anti-D IgG to all RhD-negative pregnant women despite the fact that around 35–40% of these women will be carrying an RhD-negative fetus, and these women can of course not be RhD-immunized in the current pregnancy or after delivery of their RhD-negative child.

With the discovery of cell-free fetal DNA (cffDNA) in the plasma of pregnant women, ref. [5] it has become possible to do noninvasive RhD typing of the fetus [6]. This technique is now used in several countries in Europe to identify which RhD-negative women are carrying an RhD-positive fetus [7,8,9], and these countries have changed their routine to targeted antenatal RhD prophylaxis, i.e., hyperimmune anti-D IgG is only administered to RhD-negative women carrying an RhD-positive fetus. This is reflected in several national guidelines for the care of pregnant women in many European countries [10,11,12].

Knowledge of the fetal RhD type is also of major importance for clinicians to manage the treatment of RhD-negative pregnant women who have become RhD-immunized in relation to a previous delivery of an RhD-positive child. If the mother’s anti-D levels are high, there is a need for close clinical follow-up during pregnancy, which will involve multiple ultrasonographic assessments of the fetal blood flow as a surrogate marker for fetal anemia [13]. Further, if these examinations indicate fetal anemia, the woman will need to be referred to a specialized center that will perform an intrauterine transfusion of RhD-negative blood.

## 3. Technical Considerations Related to Prediction of Fetal RhD Type

The requirements of the assay performance depends on whether the assay is used for screening RhD-negative women to guide prenatal prophylaxis or whether the assay is used for determining the fetal status in an already immunized woman [14]. The main common technical challenge when using cffDNA for determination of the fetal blood group is the low concentration of fetal DNA in the maternal circulation. Hence, the absence of an RhD signal could either be interpreted as an RhD-negative fetus, or alternatively, the fetus could be RhD positive, but the very low numbers of DNA sequences from the fetal RhD gene did not allow these sequences to be amplified, resulting in a false negative result. Consequently, it is important to ensure that the preanalytical steps such as transport, storage, centrifugation and extraction are carefully monitored and validated [14]. The use of automated DNA extraction gives a standardized process. There are several robots on the market that use magnetic beads in the process of DNA extraction. The possibility to send data from the extraction robot to the analyzing instrument and the PCR result to the laboratory information system, is a key factor for a fully automated process avoiding human errors.

A fetal marker, not present in the maternal DNA, can be used for confirmation of the presence of cffDNA in the sample. Commonly used fetal markers are hypermethylated *RASSF* [15] or, for male fetuses, Y chromosome sequences [16]. Some assays instead use extraction controls by adding artificial DNA (synthetic oligonucleotide), or DNA from another species, during the extraction process to monitor the performance [17]. Hence, if the control DNA is not giving the expected result in the polymerase chain reaction (PCR), the DNA extraction should be repeated. Other assays amplify a housekeeping gene to obtain the total amount of cell-free DNA (cfDNA) of both maternal and fetal origin, applying upper and lower Ct limits, for a valid result [8]. A low Ct value indicates high levels of maternal cfDNA, which potentially can hamper the result, whereas a high Ct value indicates that too little cfDNA has been extracted. However, studies have shown that internal controls to prove the presence of fetal DNA are not necessary if the screening assay has shown sufficiently high sensitivity [18]. Nevertheless, if the assay is used to guide treatment of an immunized women, it is crucial to avoid false negative results, and the above-mentioned precautions may therefore not be considered sufficient. For immunized women, in whom the initial test showed an RhD-negative fetus, it can be recommended to repeat testing later in the pregnancy, both to reduce the risk of human error and to allow testing when the cffDNA levels have increased, which will result in increased sensitivity [14].

The RhD-negative status is most often caused by the complete deletion of the *RHD* gene; however there are numerous *RHD* variants, which can complicate prediction of the fetal RhD type. In assays for fetal *RHD* screening, the detection of two exons provides a robust assay [7,9], but tests with only one exon do also exist [8], as well as tests where more than two exons are detected [19]. Using several exons gives the possibility to distinguish between different variants including the RhD-negative pseudogene (RHD*08N.01).

Some laboratories offer one test for antenatal *RHD* screening and another, detecting more exons, for immunized women [20]. However, in women with *RHD* variants, the fetal *RHD* type can quite often not be determined, and RhD prophylaxis is recommended as a precautionary approach. It is important that laboratories and manufactures of assays for fetal RhD typing describe the assay limitations related to variants. Unfortunately, most studies have been performed in mainly Whites, and the implementation of *RHD* screening in nonwhite populations should be carefully validated [21]. A study performed in a mixed population of pregnant women in Argentina demonstrated that the combination of exons 5 and 10 gave conclusive results [22]. Another study from Brazil also demonstrated that the fetal RhD type can reliably be predicted in a genetically mixed population of pregnant women [23]. However, they used a more complicated set up that is not practical in large-scale screening situations [23].

Realtime PCR is the most common method used for the prediction of the fetal RhD type due to its accessibility and low cost. Yet, new techniques such as massive parallel sequencing (also called next-generation sequencing, NGS) and digital PCR (dPCR) can also be used, but are more suitable for complex cases, such as when the mother is carrying a silent *RHD* allele [24,25,26].

Cord blood typing has been discontinued in countries where the antenatal *RHD* screening has been implemented for routine use [27]. This means that the postnatal prophylaxis can be given without waiting for the result of the newborn’s serological RhD status, i.e., anti-D IgG is often administered during delivery.

New legislation in the European Union will come into force in 2022 requiring manufacturers of in vitro diagnostic (IVD) assays to validate their assays according to these new rules [28,29]. Currently, many laboratories use their own in-house assays, which will need to be validated according to the same legislation or changed to a commercially available IVDR assay. At this time, there are four CE-marked kits according to the current European In-Vitro Diagnostic Devices Directive (IVDD 98/79/EC) available or just to be released on the market. Some characteristics of these assays are summarized in Table 1.

Antenatal RhD typing and targeted RhD prophylaxis have not yet been implemented in North America. One of the reasons for this is the concern related to the ability of assays based on cffDNA to reliably predict fetal RhD in multiethnic populations. Another issue is the costs of such a program. Two studies from the US and Ontario, Canada demonstrated a significantly higher costs associated with antenatal RhD typing and targeted prophylaxis [30,31], whereas another study from Alberta, Canada reached the opposite conclusion [32]. As the prices for assays for antenatal RhD typing go down, it is possible that antenatal RhD typing and targeted RhD prophylaxis will also be implemented in North America. As discussed below, such a program also has favorable ethical implications because it will prevent anti-D to be used in women who do not need this drug.

## 4. Fetal and Neonatal Alloimmune Thrombocytopenia

### Clinical Considerations

Although fetal thrombocytopenia is of transient nature, the clinical spectrum of FNAIT spans a continuum from no symptoms to petechiae, mucosal bleeding, hematomas, retinal bleeding and ICH that may result in stillbirth or a life-long disability. FNAIT is a rare disease because only around 2.1% are HPA-1a negative [33], which is a requirement for developing HPA-1a antibodies. Moreover, the propensity of developing anti-HPA-1a is closely linked to carriers of a particular HLA type. Women who are HLA-DRB3*01:01-positive have a 25 times higher risk of becoming HPA-1a immunized as opposed to those who do not carry this HLA type [34]. Furthermore, those few HPA-1a and HLA-DRB3*01:01-negative women who become HPA-1a-immunized very rarely give birth to severely thrombocytopenic children, and, likewise, ICH is extremely rare in fetuses/newborns of HPA-1a and HLA-DRB3*01:01-negative women [35]. As only around 27% of Whites are HLA-DRB3*01:01-positive [36], the proportion of pregnant women who are at risk of having a fetus/newborn suffering from FNAIT is only around 0.5%. Fortunately, ICH is rare and has been estimated to occur in 1 of 10,000 unselected pregnancies [37].

FNAIT usually turns up in a term infant like a bolt from the blue without any prior suspicions during pregnancy. In a subsequent pregnancy, the woman will be offered close clinical follow-up, and most centers will start treatment with high-dose intravenous IgG (IVIg). In this context, it is prudent to mention that (1) IVIg is used off label for this indication as the efficacy in this condition has never been tested in a placebo-controlled clinical trial; (2) there is no international consensus of whom to treat, when to start treatment and dose of IVIg [38]; (3) there are significant side effects associated with IVIg treatment [39,40,41,42]; (4) this treatment is incredibly expensive and may easily exceed USD 100,000 per treated woman; (5) there is a worldwide shortage of IVIg [43]; and (6) treatment of one HPA-1a-immunized women requires tremendous donor efforts: 4.5 man-months of plasmapheresis [38].

Thus, when consulting a pregnant woman with a previous obstetric history with anti-HPA-1a-associated FNAIT, it is essential to know if the fetus is HPA-1a-positive or negative. If the fetus is HPA-1a-negative, there is of course no reason for concern in the current pregnancy. Therefore, many centers ask for a sample from the father in order to determine if he is HPA-1a-homozygous or HPA-1a/1b-heterozygous. In the first case, the fetus will be HPA-a-positive, whereas in the second case, there will be a 50% risk that the fetus will be HPA-1a-positive and hence at risk of FNAIT. With a heterozygous father, many centers determine the fetus’s HPA-1 genotype by polymerase chain reaction (PCR) performed on DNA extracted from fetal cells obtained by amniocentesis.

FNAIT has not previously been considered for any prophylactic efforts similar to HDFN because it was generally believed that immunization against HPA-1a mostly occurred during the course of the first incompatible pregnancy. This view was based on data from retrospective studies, but two large prospective clinical studies conducted in England and Scotland, respectively, indicated that the majority of HPA-1a immunizations occur after a previous immunizing event, such as the delivery of an HPA-1a-positive child [44,45]. The results of a very large prospective clinical trial on FNAIT conducted in Norway between 1995 and 2004 confirmed this view [46]. In this study, it was shown that 75% of all cases of HPA-1a alloimmunization occurred in relation to the delivery rather than during pregnancy [47]. The realization that the majority of cases of alloimmunization occur after delivery of the first HPA-1a-incompatible infant paved the way for the idea of preventing HPA-1a immunization in the same manner as preventing RhD immunization by hyperimmune anti-D IgG. This idea was supported by preclinical studies in a mouse model of FNAIT in which it was documented that the administration of antibodies against paternally derived platelet antigens could suppress the development of a maternal antibody response against these antigens, reduce the number of thrombocytopenic pups and reduce the number of miscarriages and pups with ICH [48].

In past years, the EU-financed PROFNAIT consortium worked on the development a hyperimmune anti-HPA-1a IgG to be used as a prophylaxis against HPA-1a immunization, analogous to anti-D, which has been used successfully over the last 5 decades for the prevention of RhD immunization and HDFN [49]. Encouraging results from the phase 1/2 study were recently published [50]. If the results of the pivotal phase 3 study turn out successful, it will be possible to prevent HPA-1a immunization and FNAIT, and this may encourage national health authorities to add FNAIT screening to their antenatal health care programs. A screening program will most likely include maternal HPA-1a and HLA-DRB3*01:01 typing as well as fetal platelet typing. As the hyperimmune anti-HPA-1a IgG is manufactured from plasma collected from HPA-1a-immunized women, the supply of plasma for drug production is limited. Hence, it is essential to reserve the prophylactic drug for the HPA-1a-negative women with the highest risk of having a child with severe thrombocytopenia; i.e., those who are HPA-1a-negative (around 2.1%), HLA-DRB3*01:01-positive (approximately 27%) and who are carrying an HPA-1a-positive fetus (circa 85%).

Although the prediction of the fetal platelet type in principle could be performed by initial HPA-1a typing of the father followed by HPA-1a typing of the fetal DNA obtained from cells collected by amniocentesis in case the father was HPA-1a/b-heterozygous, this approach would be unpractical in a screening setting. As an alternative, several laboratories in Europe have developed noninvasive methods for fetal HPA-1 genotyping that take advantage of the presence cffDNA in the mother’s plasma [51].

## 5. Technical Considerations Related to Prediction of Fetal HPA-1 Type

The technical challenges associated with prenatal HPA-1a typing are considerably larger than prenatal RhD typing: In contrast to fetal RhD typing, which implies detection of sequences from the *RHD* gene in the mother’s plasma, there is only a single nucleotide difference between genes encoding HPA-1a and HPA-1b. Hence, the number of the fetal DNA strands in the plasma encoding HPA-1a is very low compared with the number of the maternal DNA strands encoding HPA-1b. This small amount of cffDNA in ‘a large ocean’ of cell-free maternal DNA represents an analytical challenge, particularly early in the pregnancy when the proportion of cffDNA is at its lowest. To address this challenge, different methods have been used to try to make fetal HPA1a typing both cost-effective and sensitive.

The first assay for fetal HPA-1 typing used a similar method with real-time PCR as for detecting the *RHD* gene. However, this was not successful without adding a pre-PCR step utilizing the restriction enzyme MspI, which recognizes and cleaves the DNA sequences encoding the maternal HPA-1b allele, hence reducing the risk of unspecific amplification [52]. The disadvantages associated with this method are the risk of incomplete enzyme digestion and the lack of internal controls for the presence of cffDNA, which could lead to a false negative result.

Another method, the coamplification at lower denaturation temperature PCR (COLD-PCR), utilizes melting temperature differences between variant and wild-type sequences, which will favor the amplification of the less abundant allele [53]. This method has been included in a workflow where first the HPA-1 status of the mother is determined by high-resolution melting (HRM) PCR, and if the mother is HPA-1-negative, COLD-PCR is applied on the same extracted DNA to determine the fetal genotype [54]. The set up shows accurate results as early as gestational week 12, but suffers from the same weakness as the previous methods since no internal control is included [54].

Novel technologies such as NGS and digital PCR are promising alternatives but involve expensive equipment not available everywhere. NGS, a technology in which millions of nucleotides are sequenced at the same time, consists of several steps: (1) DNA fragmentation, (2) adapter ligations, (3) sequencing and (4) alignment and data analysis. Currently, two main platforms using different ways of nucleotide detection are on the market. Ion Torrent^™^ (Themo Fischer, Waltham, MA, USA) uses a semiconductor chip where the pH change is detected when a nucleotide is incorporated, whereas the Illumina technology use fluorescence. Both whole genome sequencing and exome sequencing as well as targeted sequencing can be performed. Target enrichment of only selected parts of the genome is suitable for fetal genotyping because it increases the proportion of the targeted region [55]. Targeting can be done with an additional step using PCR (amplicon-based target enrichment) or probes (hybridization-based target enrichment) to capture target sequences.

The successful detection of fetal HPA-1a-positive sequences has been demonstrated in several studies using NGS [56,57]. The use of single-nucleotide variants (SNVs) for estimation of the fetal fraction works as an internal control, which makes NGS a reliable method [56]. To conclude, NGS is highly specific but time-consuming, and requires the analysis of several samples at the same time to be cost-effective.

Digital PCR (dPCR) is a sensitive technology based on PCR performed on partitions, i.e., the sample is separated in units, containing either zero, one or a few copies of the targeted DNA before PCR. After amplification, partitions containing target DNA are detected by fluorescence, and the positive and negative reactions are counted. To account for partitioning errors, a Poisson correction model is applied by the software. There are a number of different methods for separating the partitions such as microchambers, channels, printing-based sample dispersion and microfluidic technology, where the DNA is divided into water-in-oil droplets [58]. dPCR performs with high accuracy even when only a small volume of sample is available, which makes the method suitable for accurate noninvasive prenatal typing early in pregnancy [59]. Moreover, both *RASSF1a* and autosomal SNPs from the SNPforID panel have been used as internal controls with good results [59]. However, this method still has some disadvantages such as contamination risk, limited possibility for high-throughput, cost and lack of commercial assay kits. Table 2 summarizes some characteristics of the mentioned methods for HPA-1a typing.

Today, routine maternal HPA-1a typing is not used for identifying pregnant women at risk of having a child with FNAIT. Antenatal HPA-1a typing is only used for HPA-1a-immunized women at some centers in Europe. However, a hyperimmune anti-HPA-1a IgG is under clinical development for the prevention of HPA-1a immunization and FNAIT [50]. If the pivotal phase 3 clinical trial demonstrates that this drug can prevent HPA-1a immunization and FNAIT, there are reasons to believe that national health authorities will recommend that maternal HPA-1a typing be included in their antenatal health care program. In that case, there will also be a need for targeted HPA-1a prophylaxis. As HPA-1a immunization is primarily restricted to women who are HLA-DRB3*01:01-positive, it will only be necessary to administer the hyperimmune anti-HPA-1a IgG to those women who are HPA-1a-negative and HLA-DRB3*01:01-positive carrying an HPA-1a-positive fetus. NGS could be used to determine if a pregnant woman, who initially has been typed as HPA-1a negative, is at risk of having the pregnancy complicated with FNAIT. It is feasible to design one NGS assay that determines the maternal HPA-1 type (as a control of the initial screening), the maternal HLA-DRB3*01:01 status and fetal HPA-1 type. Since NGS is a very sensitive assay (Table 2), it could be applied late in the first trimester, and as explained below, an early answer regarding the risk of FNAIT is essential for the expecting mother. Thus, future inclusion of FNAIT screening in the antenatal health care program will hopefully go hand in hand with the development of tests that can determine the FNAIT risk status early in pregnancy.

## 6. Necessity of Noninvasive Prenatal Blood and Platelet Typing—Ethical Considerations

With the introduction of noninvasive prenatal testing in immunohematology, it has become possible to circumvent many ethical challenges: Many countries that have implemented antenatal RhD prophylaxis, administer hyperimmune anti-D IgG to all pregnant RhD-negative women. For 35–40% of these women, there is no risk of HDFN in the current pregnancy because they will be carrying an RhD-negative fetus. Hence, these women receive a drug for which there is no indication, which basically violates the generally accepted ethical position that a patient should not receive an unnecessary drug. Furthermore, hyperimmune anti-D IgG is a special drug as it is manufactured from plasma collected from RhD-immunized individuals. Most of these RhD-negative individuals are frequently transfused with RhD-positive RBCs in order to boost their antibody response to maximize the amount of anti-D in the plasma that is harvested from these special donors. These individuals are exposed both to the risks associated with the transfusion of RhD-positive RBCs (such as transfusion-associated infections) and the risks associated with plasmapheresis. Although both these risks are small, it is highly questionable if it can be justified to use a significant amount of the drug manufactured from plasma harvested from these special donors to patients who do not need the drug.

It also worth mentioning that while parts of the world, mainly high-income countries, are using RhD prophylaxis for women who do not need it, approximately 50% of the women around the world who require this type of immunoprophylaxis have no access to the drug. A nonprofit organization, Worldwide Initiative Rh Disease Eradication (WIRhE), has been launched to spread awareness about Rh disease and the lack of access to both blood typing and prophylaxis [60,61].

The screening programs of pregnant RhD-negative women that include noninvasive fetal RhD typing, which have been implemented in many European countries [7,8,9], have avoided the above-mentioned ethical challenges by identifying those RhD-negative women who should receive antenatal RhD prophylaxis to prevent RhD immunization and HDFN in subsequent pregnancies.

For an HPA-1a-immunized pregnant woman with FNAIT in her obstetric history, it is essential to know the HPA-1 type of the fetus. As a first step, it is common practice at many centers to do HPA-1a genotyping of the father, and if he is HPA-1a/b to continue with amniocentesis of the mother. Hence, the platelet type of the (presumed) father determines if the mother should undergo amniocentesis or not. This becomes an ethical challenge if the mother knows that her spouse is not the father. Furthermore, during the couple’s consultation with the physician, she will be put into a very difficult situation when the physician asks for a sample from her spouse! Furthermore, the increased use of assisted reproductive technology (ART) reinforces limited use of testing the spouse [62,63].

Another ethical aspect is related to the invasive nature of amniocentesis. This procedure is associated with a risk of fetal death, which should be weighed against the risk of having a severely thrombocytopenic child. Moreover, amniocentesis may also set off fetal–maternal bleeding that could boost the mother’s production of anti-HPA-1a, which in turn could worse fetal thrombocytopenia and increase the risk of severe intrauterine bleeding such as ICH.

A procedure for fetal HPA-1 determination that involves paternal HPA-1a typing, amniocentesis, expansion of fetal amniotic cells, extraction of DNA from fetal amniotic cells and finally HPA-1a typing by PCR takes a long time. This waiting time is very stressful for the expecting mother: Will the fetus be HPA-1a-positive and then at risk of FNAIT or would it be HPA-1a-negative? The latter result would be a relief for both the mother and the maternal–fetal medicine specialist, because no further follow-up during pregnancy would be necessary. Hence, it is preferable to know the fetal HPA-1 type as soon as possible after the woman has discovered she is pregnant. As explained above, some of the techniques that are used for noninvasive prenatal HPA-1 typing can be performed late in the first trimester and will minimize the waiting time for the pregnant woman. In addition, noninvasive prenatal HPA-1 typing will also avoid the other ethical challenges discussed above.

Although the results obtained from the (presumed) father should not be used for making clinical decisions for the mother, it is essential for the treating physician to know if the pregnancy is the result of ART, and if this is the case, if the conceived oocyte comes from the mother or from another woman. In the latter case, there is a risk that the fetus is homozygous for the RBC or platelet antigen to which maternal antibodies have been produced, which may increase the severity of the affected fetus. Due to the large economic and emotional costs related to these pregnancies, Curtis et al. has recommended that surrogate mothers and women who are donating oocytes for ART should be HPA-1 typed [62].

The use of noninvasive prenatal fetal RhD and HPA-1a is still limited. The reasons are both due to the technical challenges and the costs associated with these analyses. It has been argued that it is cheaper to administer anti-D to all RhD-negative women irrespective of whether or not they are carrying an RhD-positive fetus, as opposed to a program that includes noninvasive prenatal fetal RhD typing of all RhD-negative women and the administration of anti-D to only those women who need the prophylaxis.

There are reasons to believe that the use of noninvasive preclinical testing in immunohematology will increase in the near future because this will eliminate a number of ethical challenges and also because technical developments and decreasing prices will make this technology accessible to more laboratories.

## 7. Conclusions

In recent years, noninvasive prenatal blood and platelet typing based on cffDNA has increased considerably in immunohematology. Some of these analyses are used for rare clinical conditions and are technically challenging and are currently only performed in reference laboratories. By implementing this type of technology in immunohematology, a number of the ethical challenges associated with previous methods for fetal blood and platelet typing can be avoided. In the near future, the use of noninvasive prenatal testing will most probably increase as the costs of these assays decrease.

## Figures and Tables

**Table 1:** Available CE/IVD kit for fetal *RHD* genotyping with real-time PCR.

	Devyser RHD	NIMoTest^®^	FetoGnost^®^ Kit RHD	Free DNA Fetal Kit^®^ RhD
		**Fetal RHD qPCR Kit**		
Use for immunized women	yes	no	no	yes
Use for antenatal *RHD* screening	yes	yes	yes	yes
Detection of	Exon 4	Exons 5, 7	Exons 5, 7, 10	Exons 5, 7, 10
Extraction control	GAPDH	Synthetic DNA	FetoGnost^®^ Kit IPC	Maize DNA
Use from gestational week	10	11	11	9
Maximal age of sample in EDTA tube	5 days	72 h	^#^	48 h ^‡^
Need to repeat negative results	no	yes ^¤^	no	yes ^¤^
Distinction of maternal RHD*08N.01	no	yes	yes	yes
Suitable for automation	yes	yes	yes	yes

^¤^ if performed before week 16. ^#^ not stated in kit insert. ^‡^ 10 days in Streck^®^ tube.

**Table 2:** Inhouse assays for fetal HPA-1 typing.

	Realtime PCR with Digestion of Maternal Allele	Cold PCR	NGS (Targeted Massive Parallel Sequencing)	Digital PCR
Use from gestational week	18	12	13	8
Control for cffDNA	no	no	yes	yes *
Cost	low	low	high	high
Turnaround time	Medium	Medium	Long	Medium
References	[52]	[54]	[56,57]	[59]

* can be included.

## Data Availability

Not applicable.
